# Anoikis resistance and cancer

**DOI:** 10.1186/s12885-025-15178-6

**Published:** 2025-11-14

**Authors:** Steven M. Frisch, Gangqin Hu

**Affiliations:** 1 Department of Biochemistry and Molecular Medicine, West Virginia University, 64 Medical Center Drive, Campus box 9142, Morgantown, WV 26506 USA; 2 Department of Microbiology and Immunology, West Virginia University, 64 Medical Center Drive, Morgantown, WV 26506 USA

**Keywords:** Anoikis, Mechanotransduction, Epithelial extrusion, Apoptosis, Translational control

## Abstract

Normal epithelial cells detach from extracellular matrix during normal homeostasis by a selective process called epithelial extrusion. Thereafter, they undergo apoptosis, in a phenomenon called anoikis. Metastatic tumor cells, however, detach from normal epithelial tissues or primary tumors and survive in circulation, sometimes long enough to disseminate and colonize metastatic sites. The thirty years since the discovery of anoikis, exciting progress has been made in understanding anoikis mechanisms and the diverse mechanisms by which tumor cells evade or subvert them. Previous reviews have summarized earlier aspects of anoikis, including integrin-apoptosis signaling, Epithelial-Mesenchymal Transition and intracellular metabolism. Based on recently emerging work, we focus here on four issues that advance our conceptual understanding significantly: How do cytoskeleton and signaling interface so as to control anoikis? How is epithelial extrusion programmed in normal vs. cancer cells, upstream of anoikis? What is the role of translational regulation in anoikis? How do tumor cell-blood cell interactions affect anoikis? This review summarizes recent observations that inform these issues, and provides perspective on future problems to be investigated.

Anoikis is defined as programmed cell death occurring when cells lack appropriate integrin-matrix interactions, for example, in conditions of detachment. Cell-matrix detachment occurs at impressive rates to maintain homeostatic cell numbers; notably, the gastrointestinal tract lining is replaced in its entirety about every 3–5 days (depending partly upon microbiota composition). Which cells are shed from epithelia appears to be at least partially non-random: subtle cytoskeletal changes in particular cells mark them for shedding, in a physiologic process known as (epithelial) cell extrusion [1]. The ability of tumor cells to evade anoikis is crucial for metastasis - the subject of this review article.

Our laboratory (S.M.F.) discovered anoikis accidentally in the course of investigating how the adenovirus E1a protein reversed the transformed phenotype of diverse human tumor cell lines, for which a mechanistic explanation was far from reach in the early 1990s. We were not surprised to observe that tumor cell lines with E1a transgenes failed to form colonies in soft agar, i.e., were anchorage dependent. We were surprised, however, to observe that individual cells actually died under conditions of denied attachment. Concurrently, we discovered that E1a reversed the epithelial-mesenchymal transition, thus committing tumor cells of diverse lineages into well-differentiated epithelial cells. When combined, these observations suggested that epithelial cells were anchorage-dependent for survival, not just growth. Stated conversely, apoptosis was the default response to matrix detachment. Hence, we proposed the term “anoikis” (Greek, meaning “homelessness”) to describe succinctly the apoptotic response to matrix detachment. The preliminary data we published connecting anoikis-resistance to cancer was followed by an avalanche of subsequent reports supporting this concept. In fact, at this writing, about 2,800 publications about the role of anoikis in cancer have emerged–about six publications per month—since its discovery, with increasingly deep mechanistic progress as well as questions on many fronts.

This review will critically address several current issues and controversies in the regulation of anoikis in normal vs. tumor cells. In particular, we focus on the following questions:

1. How do cytoskeleton and signaling interface so as control anoikis?
2. How is epithelial extrusion programmed in normal vs. cancer cells, upstream of anoikis?
3. What is the role of translational regulation in anoikis?
4. How do tumor cell-blood cell interactions affect anoikis?

Notably, other reviews have comprehensively summarized other aspects of anoikis, discussing numerous cancer-type-specific mutations/epigenetic changes, the roles of lipid signaling, altered intracellular metabolism, redox status, EMT and death receptor signaling [2–10]. A comprehensive and current review of individual genes, proteins and pathways, stratified by tumor type and including an excellent summary of current trends in therapy has been published recently [6]. Here, we present a thematically focused discussion that complements previous reviews by establishing a framework for selected current and future areas of intensive investigation.

## The cytoskeleton controls anoikis

At its core, anoikis, and conversely, integrin-dependent cell survival, is a phenomenon of mechanotransduction from cytoskeleton to signaling pathways controlling apoptosis. This connection can be quite indirect. Integrins coordinate growth factor response pathways, leading to (1) kinase signaling downstream of integrin-dependent receptors [11], and (2) effects upon cell metabolism that impinge upon both (1) as well as mitochondria-based apoptotic signaling [12]. A primary example is the constitutively activated mutants of the epidermal growth factor receptor, which circumvent anoikis both through downstream receptor signaling cascades as well as by protecting intracellular metabolism against pro-apoptotic alterations [13]. 

Early reports demonstrated important roles for actin cytoskeleton in anoikis, utilizing such “blunt instruments” as latrunculin A or cytochalasin B, additionally indicating roles for Rac family GTPases [14, 15]. Recent reports demonstrate novel and exciting mechanistic roles of actin cytoskeleton in anoikis regulation. Specifically, structures called blebs and “Giant Unilocular Vacuoles” (GUVacs) have been implicated.

GUVAcs are large vacuoles -- constituting a major fraction of the cell volume -- that form under conditions of cell-matrix detachment (at low frequency) and/or, much more frequently latrunculin treatment [16]. Unlike endocytic vesicles, whose functions are partially actin cytoskeleton-dependent [17], GUVacs form in response to actin depolymerization. GUVac assembly requires at least two members of the septin family of proteins that regulate and interact with membrane curvatures that form under conditions of uneven cytoskeletal tension, SEPT2 and SEPT9 [18]. The septins, in turn, require PI3P phosphoinositides present in cell membranes as an activating factor (the products of PI3K enzymes). Interestingly, inhibition of GUVac assembly via diverse approaches -- including knockout of the PI3K enzymes, septins, or pharmacologic inhibition with a septin inhibitor – sensitized cells to anoikis. These observations indicated a major role for GUVacs in anoikis resistance. Whether human tumors up-regulate the GUVac pathway remains to be determined; however, septin and PI3K up-regulation in cancer have been observed [19, 20].

While the GUVac concept described above is mechanistically intriguing, several key aspects are concerning. First, the percentage of GUVac-positive cells after cell detachment alone was quite low -- higher percentages were obtained via actin inhibitor drugs – raising concerns about physiologic relevance. Secondly, the depolymerization of actin following matrix detachment alone was assumed but not shown explicitly; alarmingly, staining for polymerized actin following 24 h detachment without drug treatment was strongly detected. In the MCF10a cell line used in the study, the depolymerization of actin following matrix detachment alone is below detection (SMF, unpublished data). Additional concerns were that (1) actin depolymerization also desensitized cells to death receptor induced apoptosis, indicating a role for GUVacs in apoptosis that was not necessarily specific for anoikis; (2) to demonstrate GUVac effects on anoikis, they utilized EDTA to prevent E-cadherin mediated cell aggregation and stimulate anoikis, which may produce a number of off-target effects; (3) they were unable to show GUVac formation in several other cell lines, raising concerns about the generality of the phenomenon.

These concerns aside, there is a solid rationale to further investigate the roles of actin depolymerization, actin dissociation from the membrane, and membrane curvature/septin function in anoikis. In particular, the actin-membrane tethering component ankyrin-G was reported previously to be widely down-regulated in anoikis-resistant tumor cells that resulted from the Epithelial-Mesenchymal Transition [21]. The loss of ankyrin-G protein conferred anoikis-resistance via liberating a novel transcription factor called NRAGE, allowing it to form a complex (with TBX2) that down-regulated p14ARF expression. In turn, the loss of p14ARF – a common genetically driven event in human cancer – conferred anoikis-resistance [21]. Points of possible intersection between the GUVac pathway with the ankyrin/p14ARF pathway, under conditions of altered actin tensional dynamics, will be interesting to investigate, especially because septins are also involved in actin-membrane tethering [19].

A second cytoskeletal insight, apparently distinct from GUVacs, has recently been reported: cell blebbing. Cellular blebbing – producing small vesicular structures clinging to the external surface of the cell membrane -- is stimulated by oncogenic transformation and/or partial lack of cell-matrix adhesion [22]. In transformed cells, blebs, like GUVacs, recruit septins via membrane curvature and assemble a specialized type of signaling hub distinct from integrins that promotes tumor metastasis [22]. A recent report demonstrates the importance of blebbing as a mechanism of anoikis-resistance in tumor cells [23]. First, when blebbing was inhibited in melanoma cell lines, using wheat germ agglutinin or a topological barrier (“coffins” generated by Vitrogel), anoikis was augmented in detached melanoma cells. These treatments also enhanced apoptosis in response to Raf-inhibitor drugs, indicating that core apoptotic mechanisms were likely to be affected. Septins localized to the blebs, and were functionally important, because dominant negative Sept2 or the inhibitor forchlorfenuron also produced anoikis-sensitization. Nras (which was mutationally activated in these cells) was found to interact with Sept2, and signaling downstream of Nras, using ERK nuclear localization and activation of Akt as surrogate markers, was found to be Sept2- and bleb-dependent. This suggested that Nras-induced tumor progression was at least partially dependent upon bleb-related signaling hubs. Sept2 also interacted with CD44, a cell adhesion receptor (for hyaluronan); interestingly, CD44 was shown elsewhere to regulate anoikis [24]. Finally, when blebbing, which is normally transient, was prolonged in detached cells via inhibition of clathrin-mediated endocytosis, cells were more resistant to anoikis. In summary, this report linked cytoskeletal/tensional signaling with conventional signal transduction generating anoikis resistance via cell blebbing. This was consistent with, and provided a re-interpretation of, earlier reports linking Rho activation—which is required for cell blebbing – with anoikis-resistance [25]. Moreover, the concentration of activated and wild-type Ras oncoproteins by partitioning is important for oncogenic transformation, and may now be understood in terms of bleb-mediated clustering [26]. In a larger sense, this report informs a new understanding of the important, previously established role that cell blebbing plays in tumor cell invasion and metastasis [22].

**Fig. 1:**
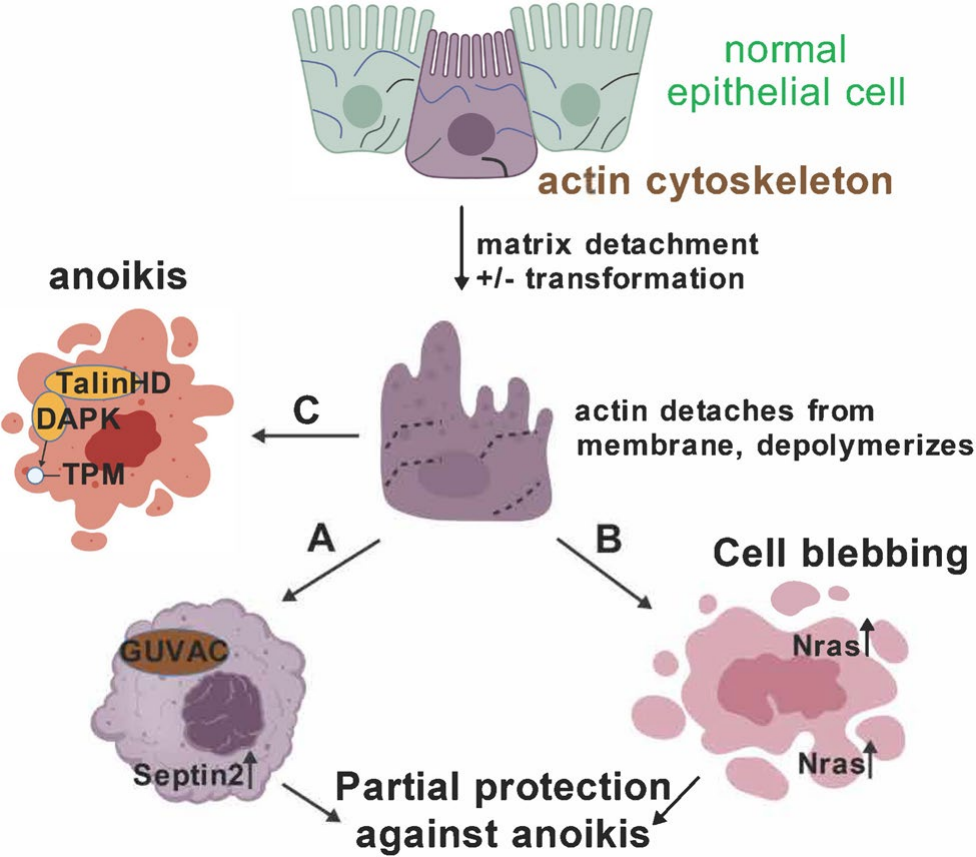
Cytoskeletal alterations both promote and suppress anoikis. Following cell matrix detachment by extrusion from a normal epithelial monolayer (or primary tumor, not shown), actin cytoskeleton detaches from membrane and perhaps depolymerizes in some cases. Two downstream mechanisms for anoikis protection may partially counteract death signaling. The first (**A**) is the formation of Giant Unilocular Vesicles (GUVACs); survival signaling downstream of GUVACs is not yet understood. The second (**B**) is cell blebbing, which is promoted by detachment and/or oncogenic transformation. Cell blebs concentrate signaling molecules such as activated Nras signaling modules, promoting their activity and promoting cell survival. **C** In detached cells, the cytoskeletal protein talin is partially cleaved and the head domain associates with DAPK, activating it. DAPK phosphorylates tropomyosin and numerous other target proteins that promotes cell death

An important but under-represented component that may link integrin signaling, cytoskeletal dynamics, cell blebbing and anoikis is a serine kinase, Death-Associated Protein Kinase (DAPK). DAPK is a tumor suppressor gene in diverse cancer types [27]. DAPK activity is activated by cell-matrix detachment or attachment to “soft” matrices, in part by calmodulin/PP2A-mediated dephosphorylation—i.e., DAPK is a rigidity mechanosensory—actually, a component of a complex multicomponent cytoskeletal sensor [28]. While DAPK substrates are numerous, the overall effects of DAPK activation are the suppression of integrin activation, with subsequent loss of cell-matrix adhesion, and paradoxically, both cell blebbing and anoikis [28, 29]. One validated substrate is tropomyosin1 (TPM1); restoration of TPM1 activity confers anoikis-sensitization in some cancer cell lines [30]. The phosphorylation of TPM1 by DAPK is involved in sensitizing cells to anoikis, thus conferring cell death on soft matrices [29]. On soft matrices, the pro-apoptotic activity of DAPK was augmented by its association with the talin head domain resulting from calpain cleavage – an event occurring normally by the core rigidity sensor—whereupon DAPK dissociates from adhesion complexes and is activated by dephosphorylation PTPN12, a cancer relevant tyrosine phophatase [29]. Conversely, tyrosine phosphorylation of DAPK by activated Src, EGFR or HER2 were found to inactivate the enzymatic activity and pro-apoptotic activity of DAPK, informing a novel mechanism for the pro-survival/pro-metastatic effects of these kinases. These studies are exciting because they link cell signaling mechanisms directly to cytoskeletal mechanosensory apparatus that regulates anoikis, an area of intensive current and future investigation. Cytoskeletal aspects of anoikis control that are discussed above are summarized in Fig. 1.

Looking toward future perspectives on the role of mechanosensing/mechanotransduction in regulating anoikis, it may prove important to consider the substantial role of autophagy. In general, autophagy is a major cell survival protective mechanism for tumor cells, and that protection includes anoikis-resistance [31, 32]. Mechanistic interplay between autophagy and anoikis - emphasizing the role of mitophagy of damaged mitochondria, the recycling of core apoptotic components, effects on ATF4-mediated transcription and mTOR-driven metabolism - has been reviewed elsewhere [31, 32]. Nevertheless, there is another newly emerging aspect of this connection, namely, the cytoskeletal effects of autophagic processes that may well impinge upon anoikis downstream. Extensive interactions among adhesion regulators (e.g., Src), structural components of adhesion complexes (e.g., paxillin) on the one hand with autophagic machinery components on the other (e.g., various ATG proteins, NBR1), regulating focal adhesion dynamics and cytoskeletal remodeling. Autophagy component proteins also affect regulate the endosomal processing of integrins and FAK-src dynamics at focal adhesions so as to promote anoikis-resistance [32, 33]. Further investigation of the connections between autophagy and anoikis in terms of cytoskeletal processes is warranted.

## Epithelial extrusion is the precursor for anoikis

The homeostasis of epithelial tissues is universally a dynamic balance between cell shedding and cell replacement. An emerging body of recent data indicate that the cells ejected from epithelial tissues are not necessarily selected randomly. Instead, a complex interplay of signaling (both intra- and extra-cellular), cytoskeletal changes and mechanotransduction processes causes specific cells to be ejected, a phenomenon called *epithelial extrusion* (reviewed in [1, 34]. Under normal circumstances, cell extrusion occurs apically, and the ejected cell is deleted via anoikis or other cell death stimuli [35]. Apical extrusion evades an inflammatory response that would otherwise accompany the release of ATP during in-residence apoptosis [36]. In the case of a tumor cell that has not yet developed anoikis-resistance, apical extrusion is thus a tumor suppressive mechanism, for which the name Epithelial Defense Against Cancer (EDAC) has been proposed [37]. Consistent with this, selective inhibition of apical extrusion leads to neoplasia in mouse models [38]. Tumor cells frequently acquire basal extrusion, providing a matrix- and growth factor-rich environment for tumor formation. In the case of tumor cells that have already acquired anoikis resistance, however -- through diverse mechanisms– cell extrusion contributes to tumor progression (via basal or apical ejection). This raises the hitherto unasked question: does the epithelial extrusion mechanism discriminate between anoikis-sensitive vs. anoikis-resistant cells? If so, how? Here, we discuss selected mechanisms regulating the extrusion of normal vs. tumor cells, because these mechanisms directly determine which cells undergo anoikis, or, conversely, metastasize.

Under normal homeostatic circumstances, epithelial cells are marked for extrusion by mechanotransduction through the stretch-sensitive receptor, Piezo1 [34, 39]. Downstream signaling upregulates the synthesis of sphingosine-1-phosphate (S1P), a major vascular permeability regulator, a suppressor of innate immunity through Toll-like receptors and a potential regulator of apoptosis in epithelial tissues. Through S1P’s G-protein coupled receptors, SIP1 induces a hypercontractile state through Rho family GTPase activation, inducing the formation of a unique “contractile ring” of actomyosin that marks the cell for extrusion [34]. Interestingly, an alternative pathway is that apoptotic cells can activate Rho GTPases directly through caspase-mediated partial cleavage of p115RhoGEF, thus stimulating the same extrusion pathway. Neighboring cells, through unknown mechanisms, reorganize their microtubule cytoskeleton, forcing the pre-extruded cell to concentrate its actomyosin ring basally, thus driving apical extrusion. Mutations in the APC tumor suppressor, bearing a microtubule-interating domain, disrupts this latter process, facilitating basal extrusion [40]. Interestingly, various Rho family genes and Rho-kinases are over-expressed oncogenic drivers in diverse cancer types [41]. This raises a novel question: does cell-intrinsic Rho activation drive the extrusion of transformed cells from epithelial tissues, thus contributing to metastasis? This will be of interest to test experimentally.

In addition to the Piezo/S1P/Rho/Actin mechanism discussed above, a novel extrusion mechanism occurring in RasV12-transformed cells has been reported [42]. An IP3 receptor-driven calcium wave initiated in the transformed cells is propagated across at least sixteen neighboring cells via successive activation of their calcium-sensitive TRPC channels. While detailed mechanisms downstream of this are unclear, the calcium waves cause the migration of neighboring cells toward the transformed cell, actin reorganization and apical cell extrusion of the transformed cell. RasV12 has separately been reported to selectively compromise apical and augment basal extrusion of transformed cells, by depleting S1P pools [43]. Curiously, apoptotic cell extrusion is proposed to use a similar mechanism. Cell blebbing is prominent in both transformed and apoptotic cells. In this connection, an important role for lamellipodia in cell extrusion has been reported [44]. A subset of lamellipodia and bleb features overlap, and both are driven by activated Rho GTPases, as is actomyosin contractility [45]. This raises the question of a potential role of blebbing prior to extrusion, that remains to be addressed.

**Fig. 2:**
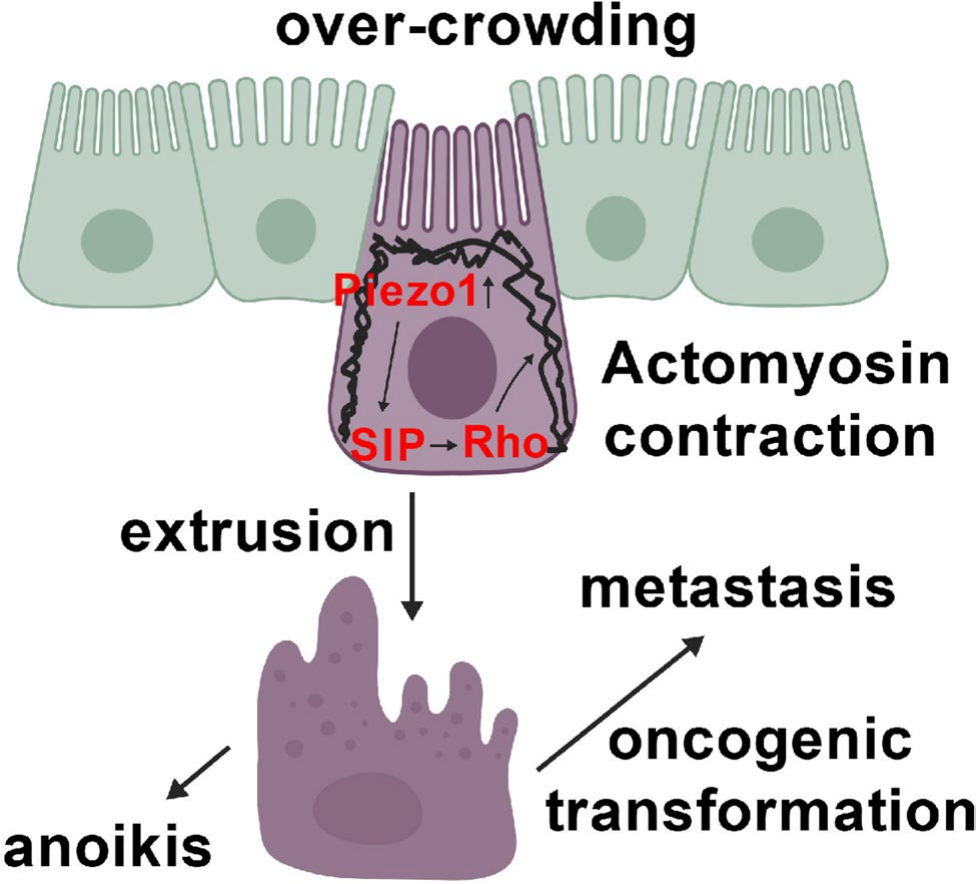
Epithelial extrusion precedes anoikis. Under normal homeostatic conditions, epithelial overcrowding results in a cell that is targeted for extrusion. This occurs in response to activation of the stretch-regulated receptor, Piezo1, which signals so as upregulate the synthesis of S1P. S1P then directly hyperactivates Rho proteins, resulting in the formation of an actomyosin ring followed by extrusion. This is followed by anoikis in normal cells or potentially metastasis in tumor cells, depending upon cell genotype and phenotype

In summary, the control of epithelial cell extrusion in normal and tumor cells is important to consider in order to comprehend the larger context under which anoikis occurs under physiologic or pathogenic conditions. The regulation of epithelial extrusion is summarized graphically in Fig. 2.

## Translational regulation plays an important role in anoikis

The selective alterations in the control of specific mRNAs into proteins plays a crucial, and, perhaps, under-appreciated role in cancer [46]. This can be understood because translation rather than transcription tends to be generally rate-limiting for rapid induction of protein expression; i.e., for many genes, a two-fold increase in protein expression requires a 1,000-fold increase in transcription or a two-fold increase in translation [47]. Accordingly, the mRNAs containing extensive 5’ secondary structures include the oncogenes C-MYC, BCL-XL and VEGFA, and the factor that both binds 5’ CAPs and alleviates these structures so as to enable translational initiation, eIF2E, is upregulated widely in cancer [48]. This is the pivotal mechanism by which the nutrient sensor/integrator MTOR renders protein translation sensitive to nutrient status, growth factor signaling and certain oncogenes [46]. The eIF4E inhibitor proteins and eIF4E itself are inactivated and activated, respectively, by MTOR. Notably, the factors mentioned here are each implicated in the control of anoikis, demonstrating the relevance of translational control [49–53].

Translational control is also predominant and rapid in response to cell stress [48] of which cell-matrix detachment is a prominent stimulus—and this response is frequently altered in cancer cells [48]. The cell stress concept provides a framework for understanding anoikis control.

The transcription factor ATF4 up-regulates cell survival response genes (e.g., anti-oxidant, DNA repair, autophagy, and anti-apoptotic genes). In response to various cell stressors, including hypoxia, endoplasmic reticulum stress, amino acid deprivation, heme deprivation or viral infection involving double-stranded RNA, the translation initiation factor eIF2a is inactivated by phosphorylation; this cell protective effect is known as the Integrated Stress Response (ISR) [54]. This effect attenuates global translation dramatically. Counter-intuitively, this inactivation also blocks the translation of an upstream open reading frame in ATF4 mRNA, permitting a noncanonical translational initiation from an internal start site to occur, rapidly generating ATF4 protein and its ensuing protective effects described above. ATF4 also represses the transcription of the p14ARF tumor suppressor, which would be predicted to protect against anoikis also [21, 55].

Interestingly, the ISR is activated in response to cell-matrix detachment, and the ensuing ATF4 accumulation protects cells partially against anoikis, counteracting, to various (context-dependent) extents, the pro-apoptotic effects of detachment [56]. In this study, the ER misfolded protein response kinase PERK was shown to be activated by cell-matrix detachment. The important role of PERK in anoikis was demonstrated by assaying for mammary epithelial cell acinus formation in three-dimensional culture, which showed increased apoptosis in PERK-knockout cells. Conversely, overexpressed PERK caused excessive luminal cell survival in acini; similar results pertaining to apoptosis were obtained in mice with mammary-specific PERK knockouts. Downstream of the ISR, increased autophagy and anti-oxidant gene expression promoted anoikis-resistance. Accordingly, PERK overexpression was shown to promote tumor progression by preventing oxidative DNA damage [57]. This report was the first to confirm suspicions that cell detachment may affect translational responses, affecting anoikis. An additional connection with the ISR is that the Epithelial-Mesenchymal Transition (EMT) – which universally programs anoikis-resistance by diverse mechanisms—also stimulates ISR signaling, a potential novel mechanism for anoikis protection [58–60].

Translational profiling of normal, non-metastatic tumor vs. metastatic tumor cells implicates translational reprogramming by MTOR as a driver of prostate cancer invasion and metastasis [61]. MTOR has context dependent effects on anoikis via autophagy and regulation of multiple biosynthetic pathways, thus affecting levels of the protective metabolite, NADPH. Detached cells, whether normal or transformed, activate the nutrient sensitive kinase AMPK; this activation is, however, an effective inhibitor of MTOR and protein synthesis in transformed – but not in normal cells – tested in this study. Accordingly, MTOR *inhibition* confers an anoikis-protective effect – in detached transformed cells, but not normal cell lines [62]. A second downstream effect of MTOR *activation* can, paradoxically, protect against anoikis, however. A particular isoform of the transcription factor CEBP-b, called LIP, is generated by translation initiation from an internal start codon within the CEBP-b mRNA; overexpression of this isoform is thought to occur and perhaps promote tumor progression [63, 64]. Activation of IGFR/Akt/MTOR signaling induced LIP protein translationally. By mechanisms that remain to be determined, LIP conferred anoikis-resistance. This paradox indicates that the effects of the MTOR pathway on anoikis are context-dependent and warrant a further examination of the role of LIP; in this connection, CEBP-b proteins form heterodimers with ATF4 proteins to activate transcription, a potential mechanism in light of the ATF4 features discussed above [65]. Another protective effect of MTOR activation may occur indirectly: MTOR, via the downstream MNK1/2 kinases, enhances the eIF4E-driven translation of SNAIL proteins, thus driving EMT and its ensuing protective effects [66]. Finally, MTOR is not only an important regulator of eIF4E activity (discussed above) but also eIFa. Under detached conditions, MTOR is inhibited, with downstream effects including the activation of the ISR pathway kinase GCN2, leading to increased phosphorylation of eIFa; this effect promotes the ISR-ATF4 pathway (elucidated above)—another protective cell survival effect of MTOR that partially counters the death signaling occurring in detached cells [67]. In summary, the effects of nutrient-sensing and stress-sensing via the MTOR pathway can affect anoikis positively or negatively depending upon the phenotypic program of the cell before vs. after detachment – a cautionary message relating to the use of therapeutic MTOR inhibitors.

The role of reactive oxygen species (ROS) in promoting apoptosis in general, including anoikis, is well established, as is the ability of antioxidant enzymes to protect against anoikis, thus promoting metastasis [68]; conversely, one countermeasure for cells to evade death is to upregulate antioxidant enzymes. The former phenomenon may well explain the lack of benefit of antioxidants in preventing tumor progression that is frequently observed [69]. Accordingly, the mitochondrial sirtuin SIRT3 transcriptionally upregulates the expression of the antioxidant gene SOD2 under detached conditions. Moreover, overexpressed SOD2 enhanced ovarian cancer cell survival under detached conditions and metastatic capability [70]. More recently, an additional selective translational upregulation of SOD2 was reported [71]. Polyribosome profiling (“Riboseq”) of attached vs. detached cells demonstrated this mRNA selective upregulation (as well as other hits remain to be investigated). The stress-responsive RNA binding protein HuR, which was previously implicated in translational regulation as well as alternative splicing, was found to accumulate in the cytoplasm of detached cells and promote the translation of SOD2 mRNA. Interestingly, the stress responsive kinase p38 MAPK was activated by detachment and it promoted the HuR-mediated increase in SOD2 translation. This kinase had previously been implicated in anoikis through the activation of Bax [72]; the related kinase Jun-N-terminal Kinase (JNK) translationally upregulates BH3-only proteins to promote anoikis [73]. Both kinases have also been reported to affect protein translation selectively, underscoring the importance of stress-responsive kinase effects on translation as a potentially important theme in anoikis control [73–75].

A recent report describes a novel eIF2E/MTOR-independent mode of cap-dependent translational initiation that affects tumor metastasis and anoikis [52]. The core of this novel mechanism is an eIF4G homologue called DAP5, which, in a complex with the factor eIF3d, can effectuate cap-dependent initiation of translation. DAP5 silencing resulted in the translational down-regulation of a subset of mRNAs, identified by assigning Translational Efficiency (TE) scores. Gene set enrichment analysis of the TE scores indicated that DAP5 selectively up-regulated genes involved in cell survival, cell migration/invasion -- including multiple integrin alpha-subunits– and EMT pathways --including the EMT-transcription factors, e.g., Snail1/2, ZEB1, Twist, as well as the EMT-associated genes encoding vimentin and N-cadherin. DAP5 knockdown did not affect eIF2E activity or ISR signaling. Furthermore, DAP5 silencing attenuated anoikis-resistance in breast cancer cells with an EMT phenotype, and, accordingly, diminished tumor metastasis but not primary tumor growth in tumor bearing mice; interestingly, the survival of tumor cells in the metastatic niche required DAP5 expression. Finally, high DAP5 expression strongly correlated with the incidence metastasis in breast cancer. In summary, the novel translation factor DAP5 may regulate anoikis directly, through mechanisms that remain to be discovered, or indirectly, through the promotion of EMT. In either event, it will be important to understand the details of how these effects are mediated so as to regulate the anoikis-resistance that accompanies EMT in tumor cells [58].

While a few notable translational switches controlling anoikis have been identified as noted above (MTOR/eIF4E, DAP5, ISR signaling, HuR/SOD2), the role of translational regulation remains to be investigated for several reasons. Target mRNAs of the stress- and nutrient sensitive MTOR/eIF2E pathway that are relevant to anoikis have been rather comprehensively identified (see above). Additional translational targets of ISR/phosphorylated eIF2a that have important effects on anoikis – besides ATF4—remain to be identified, however. In this connection, switching of Open Reading Frames (ORFs), the mechanism of ATF4 mRNA translation, is an important facet of translational regulation more broadly [76]. Many other translation factors besides eIF2a regulate ORF switching, including the stress responsive factors DEAD/H helicases, eIF4B or IRES-transacting factors (ITAF) that promote internal ribosome initiation site usage [75]. Moreover, the m^6^A methylation of adenosines in mRNAs powerfully affects their translation, alternative splicing and ORF switching in some instances; the expression of the relevant methyltransferases and demethylases are frequently regulated by EMT and/or cell stress [77, 78]. In addition, cell detachment acutely increases ROS levels and EMT suppresses them, with potential effects on translational control [78]. The Hippo/YAP/TAZ signaling pathway – established firmly as a mechanoregulator of transcription with a pivotal role in anoikis—can selectively regulate translation as well, by MST1 kinase-mediated phosphorylation of eIF4E [79, 80].

A recent report has revealed that enforced cell migration through a synthetic microporous barrier confers anoikis-resistance through a translational mechanism that up-regulates Inhibitor of Apoptosis (IAP) family proteins [81]. Cell shape changes altered by experimental “confinement” affected anoikis, as well as cell invasion and metastasis and some aspects of EMT related gene expression. While the detailed mechanism has yet to be established, this report is a cutting edge stimulus motivating further investigation of the mechanotransduction mechanisms that regulate anoikis via translational control, a core mechanism that is difficult to address experimentally.

**Fig. 3:**
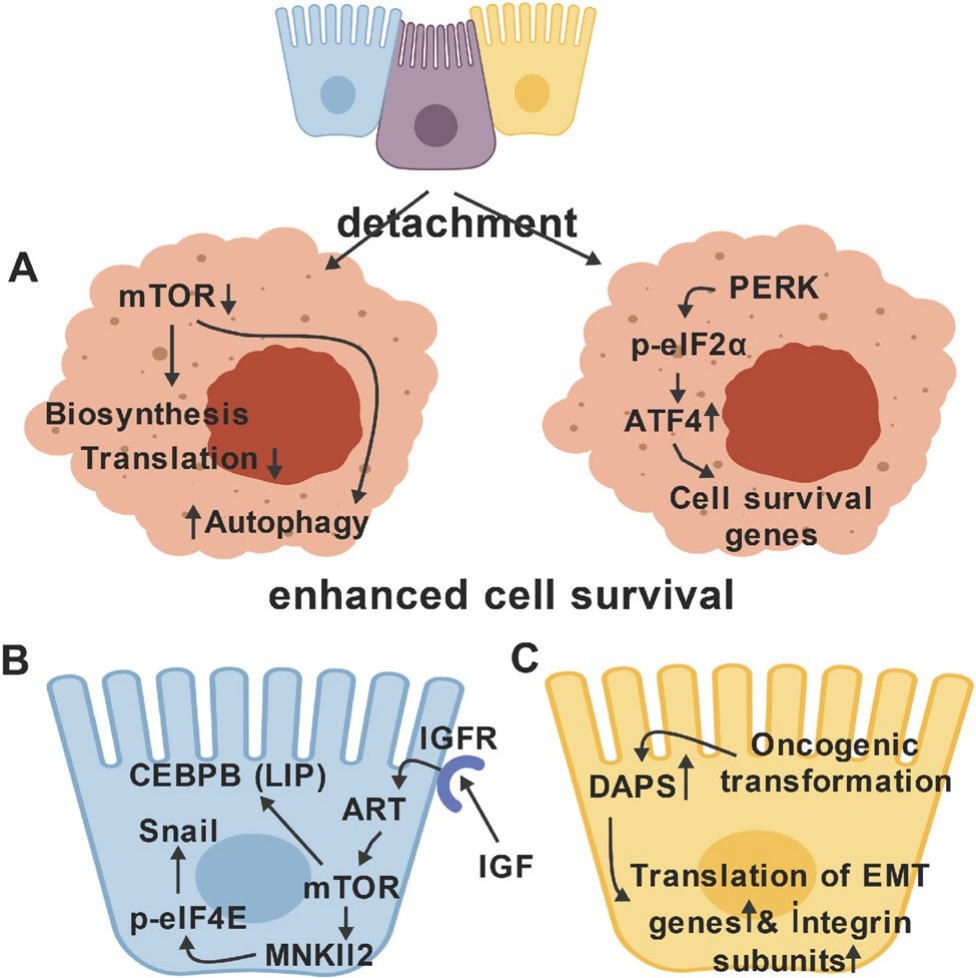
Translational alterations that help protect cells against anoikis. **A** Detachment from matrix activates the Integrated Stress Response through PERK, globally downregulating translation but selectively upregulating the translation of a subclass of mRNAs. The latter encodes the transcription factor ATF4, which powerfully induces protective genes that encode antioxidants, anti-apoptotic proteins and autophagy components. Autophagy is also promoted by the inactivation of MTOR. **B** Activation of MTOR through IGF/IGFR/Akt signaling also protects cells proactively against anoikis via MNK1/2-mediated activation of eIF4E – causing the translational upregulation of the EMT-driving factor Snail, and by selectively promoting the translation of the CEBP-b isoform LIP, with additional cell survival gene transcription ensuing. **C** A recently described Cap-dependent initiation factor, DAP5, is upregulated by oncogenic transformation, selectively promoting the translation of integrin-alpha subunit and EMT factor mRNAs

In summary, these observations indicate that much work remains to address the mechanisms by which anoikis is regulated at the translational level. The relationships among translational control, matrix engagement and anoikis are summarized in Fig. 3.

## How do tumor cell-blood cell interactions affect anoikis?

Epithelial extrusion (discussed above) destines specific cells for detachment followed by anoikis, or detachment followed by “circulating tumor cell” (CTC) status depending upon the genotype and phenotype of the targeted cell. It should be noted that tumor cells can be programmed in the primary tumor microenvironment to become anoikis resistant (reviewed [9, 82]. For example, Tumor-Associated Macrophages (TAMs) can secrete IL-6, which induces EMT in primary tumor cells through JAK2/STAT3 signaling, yielding anoikis-resistant CTCs [83]. Another important determinant of which fate applies to the extruded cell is its interactions with other cell types in the blood, which we now discuss. Notably, while this discussion is limited to anoikis, tumor cells also can be deleted by shear stress, immune cell clearance and other factors; these are discussed comprehensively in a previous review [84]. This is important to keep in mind, because in many instances, adaptations that protect tumor cells against immune attack may also protect against anoikis, if, for example, core apoptotic machinery components are down-regulated.

CTCs interact with diverse hematopoietic cells, including NK cells, T-cells, monocytes/macrophages, dendritic cells, neutrophils, and platelets, in addition to forming tumor cells homo-clusters and hetero-clusters composed of two or more cell types. The best characterized of these interactions with regard to anoikis is the CTC-platelet interaction [85]. CTCs can activate platelets through the ligand CD97 present on the CTC surface and integrin signaling on the platelet [86]. Platelets protect CTCs against anoikis by several mechanisms. One mechanism involves enhancement of tumor cell adhesion signaling and physical deformation of CTCs by “microthrombi” formed by CTC-platelet interactions [87]. Speculatively, cytoskeletal deformation could affect anoikis via transcriptional or translational effects of the Hippo/YAP/TAZ pathway, previously shown to play an important role in anoikis [79, 80]. Additionally, deformation by platelet interaction could potentially reprogram translation, promoting cell survival through IAP proteins as mentioned above [81].

**Fig. 4:**
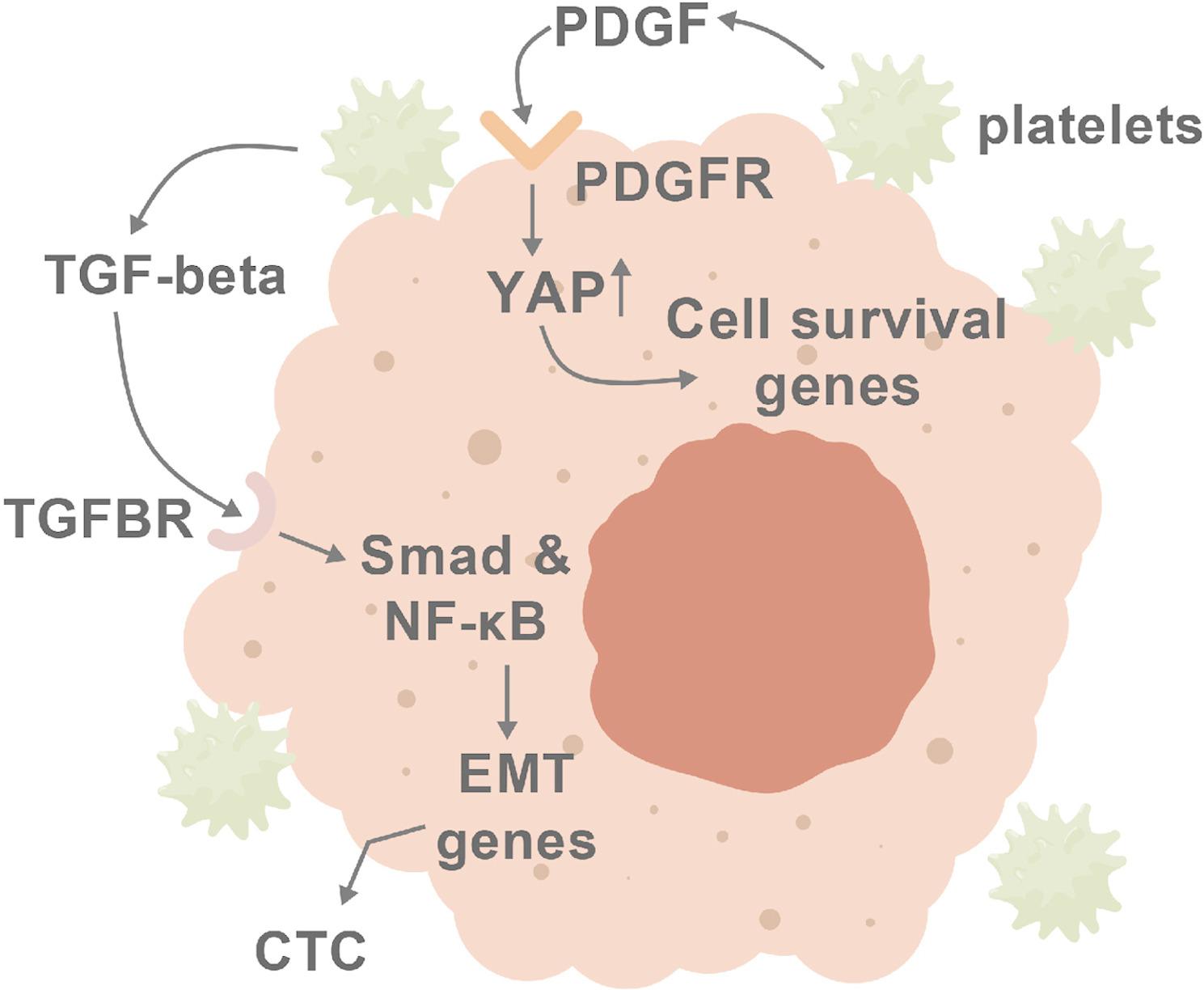
Platelets can confer anoikis-resistance in circulating tumor cells (CTC). CTC form clusters with platelets that frequently also include other hematopoietic cell types. Both by direct interaction and indirectly, through secretion of PDGF and TGFβ, several pro-survival transcriptional programs are induced, involving Smads, NF-kB and YAP

PDGF-BB and perhaps other factors from platelets confers anoikis-resistance [88, 89]. Interestingly, this effect was mediated by Rho-dependent activation, via MYPT-PP1 dephosphorylation, of the Hippo pathway coactivator of transcription, YAP; chemical inhibition of Hippo/YAP signaling (with the compound verteporfin) partially reversed the anoikis-resistance [88, 89]. This is particularly interesting in light of both the transcriptional and translation effects of Hippo/YAP signaling mentioned above, which are now shown to be responsive to signals from platelets. In addition to PDGF, activated platelets also release TGF-b, a powerful (even if transient) driver of EMT and ensuing anoikis-resistance that signals through both Smad and NF-kappaB transcription factors, which have themselves been implicated in anoikis-resistance [90–92]; direct platelet-CTC interactions contribute to these effects as well. These observations also raise the intriguing question of whether platelet-targeting anticoagulant drugs could aid in the suppression of anoikis-resistance and metastasis [93]. The control of anoikis by platelets is summarized graphically in Fig. 4.

The effects of tumor cells’ interactions with other hematopoietic cell types are less extensively characterized. It is worth noting, however, that IFN-g-secreting immune cells (e.g., T-cells, NK cells) may potentially induce anoikis-resistance through a novel mechanism. IFN-g induces a gene called G1P3 that promotes cell survival generally and protects against anoikis in particular. This effect was attributed to G1P3’s ability prevent the up-regulation of the BH3-only protein Bim after detachment, the latter protein being an important component of the anoikis mechanism [94, 95]. Finally, heterotypic clusters of tumor cells with various hematopoietic cell types may potentially affect tumor cell integrin signaling or other cell adhesion molecules with ensuing effects on anoikis.

## Future perspectives

Within each topic area discussed above lies not only a wealth of information but key questions remaining to be investigated. we consider the following points to be a non-exhaustive list of key questions in this latter category.

--What other forms of stress signaling are induced by detachment, aside from the PERK-mediated Integrated Stress Response, and how does this signaling regulate the protein translation of specific mRNAs under detachment conditions?
--Is the MTOR pathway the only pathway for sensing of nutrient status that controls anoikis? Are other nutrient sensors involved?
--Are SOD2 and ATF4s the only protective genes upregulated translationally by detachment? Or are there other important ones?
--How is the selectivity of epithelial extrusion affected by the tumor cell genotype/phenotype? By the tumor microenvironment?
--How does inflammatory signaling affect anoikis in tumor cells?

## Data Availability

No datasets were generated or analysed during the current study.
